# Limits to tDCS effects in language: Failures to modulate word production in healthy participants with frontal or temporal tDCS

**DOI:** 10.1016/j.cortex.2016.10.016

**Published:** 2017-01

**Authors:** Samuel J. Westwood, Andrew Olson, R. Chris Miall, Raffaele Nappo, Cristina Romani

**Affiliations:** a Aston University, Life & Health Sciences, Birmingham, UK; b Behavioural Brain Sciences Centre, School of Psychology, University of Birmingham, UK

**Keywords:** tDCS and language tasks, tDCS and picture naming, tDCS in control participants, Interference effects and tDCS

## Abstract

Transcranial direct current stimulation (tDCS) is a method of non-invasive brain stimulation widely used to modulate cognitive functions. Recent studies, however, suggests that effects are unreliable, small and often non-significant at least when stimulation is applied in a single session to healthy individuals. We examined the effects of frontal and temporal lobe anodal tDCS on naming and reading tasks and considered possible interactions with linguistic activation and selection mechanisms as well as possible interactions with item difficulty and participant individual variability. Across four separate experiments (*N*, Exp 1A = *18*; 1B = *20*; 1C = *18*; 2 = *17*), we failed to find any difference between real and sham stimulation. Moreover, we found no evidence of significant effects limited to particular conditions (i.e., those requiring suppression of semantic interference), to a subset of participants or to longer RTs. Our findings sound a cautionary note on using tDCS as a means to modulate cognitive performance. Consistent effects of tDCS may be difficult to demonstrate in healthy participants in reading and naming tasks, and be limited to cases of pathological neurophysiology and/or to the use of learning paradigms.

## 1 Introduction

Transcranial direct current stimulation (tDCS) is a popular technique for modifying cognition using a weak electric current. Over the past decade, thousands of articles have reported beneficial effects especially in language tasks in participants with healthy (Prehn & Flöel, 2015) and pathological brains (for aphasia, see de Aguiar, Paolazzi, & Miceli, 2015; for dyslexia see, Heth & Lavidor, 2015). Based on early research on the motor cortex, cortical excitability can be modulated via shifts in resting membrane potentials, resulting in hypopolarization/excitation versus hyperpolarization/inhibition depending on the polarity of stimulation (i.e., anodal versus cathodal). However, cognitive effects are far more complex and unpredictable (Horvath, Forte, & Carter, 2015a). This is in part because tDCS effects interact with ongoing cortical activity (see Silvanto, Muggleton, & Walsh, 2008), as indicated by the general effectiveness of tDCS in patient samples (for review, see Cappon, Jahanshahi, Bisiacchi, Turner, & Paul, 2016; de Aguiar et al., 2015). It may therefore be that tDCS can modulate cognition in pathological brains where excitability or processing capacity is unusually low or dysfunctional, but not in healthy brains where neuronal excitability is operating at optimal levels. If true, this will limit the applicability of tDCS. We aimed to gather further evidence on this question by focusing the effects of single-session, anodal tDCS in normal participants coupled with picture naming and reading tasks, and by considering the moderating influence of cortical excitability resulting from individual differences and task demands.

The reliability of tDCS in cognitive tasks has been questioned in recent reviews. Horvath et al. (2015a) found no evidence of any cognitive effects across eighty studies on healthy participants using single sessions of tDCS. In a companion review, Horvath, Forte, and Carter (2015b) also showed no *neurophysiological* effects of tDCS beyond the modulation of motor evoked potential (MEP) amplitudes. Meta-analyses focusing on working memory/short-term memory effects in healthy samples reported similarly significant but small effects of anodal tDCS (e.g., Brunoni and Vanderhasselt, 2014, Hill et al., 2015). For example, Dedoncker, Brunoni, Baeken, and Vanderhasselt (2016) found a significant but unimpressive reduction in response times following single sessions of anodal (or excitatory) tDCS applied to the left dorsolateral prefrontal cortex in healthy volunteers (effect size: −.10). However, a recent and arguably more comprehensive review by Mancuso, Ilieva, Hamilton, & Farah (2016) focusing on the effects of anodal tDCS in healthy participants revealed that effects became non-significant after correction for publication bias. This is important given the notorious “file-drawer” tendency to favor publishing studies reporting significant results.

Only one published review has examined effects of tDCS on language tasks in healthy participants, and it has not included naming tasks. Price, McAdams, Grossman, and Hamilton (2015) examined effects in verbal fluency (*N* = 6) and word learning (*N* = 2) and found a small anodal tDCS improvement in *accuracy* scores when all studies were pooled together, but also when analyses were limited to the four studies using offline stimulation (i.e., applied prior to task performance) or the three studies measuring offline effects in verbal fluency. Here as well, however, effects were small (<∼.05), and depended largely on two studies with abnormally large effects (∼.8; Flöel, Rösser, Michka, Knecht, & Breitenstein, 2008; ∼1.2; Cattaneo, Pisoni, & Papagno, 2011). What is worse, the effect in one of these studies (i.e., Cattaneo et al., 2011) has not been replicated since (see Penolazzi, Pastore, & Mondini, 2013; Vannorsdall et al., 2016; but see Cattaneo et al., 2016 for response). Another review by Jacobson, Koslowsky, and Lavidor (2012) showed no cathodal-induced decrements for language studies (0 out of 5 studies), but significant anodal-induced improvements (7 out of 8 studies). This review, however, included both patient and control samples. Moreover, since the aim was comparing cathodal and anodal stimulation, for each study, only the most significant effect for either cathodal or anodal stimulation was included across conditions, a zero effect size was assigned to null outcomes, and any effect that contradicted an anodal-excitation/cathodal-inhibition outcome was excluded. In actuality, across the four studies investigating language production in healthy participants, only 3 out of 26 effects were significant.

Variation in tDCS outcomes may be due to methodological differences across studies, especially in terms of the parameters of the applied current (for further discussion, see Antal, Keeser, Priori, Padberg, & Nitsche, 2015; Horvath et al., 2016, Nitsche et al., 2015), but also to interaction with ongoing cortical activity (see Miniussi, Harris, & Ruzzoli, 2013). Picture naming could be an important task to assess these interactions. Naming involves both the need for cortical excitation to allow retrieval of target representations and the need to curtail excitation of related words that may otherwise reach ‘activation threshold’ and be produced in error (for similar argument, see Miniussi et al., 2013). Depending on the task, one can have a relatively greater need of activation/excitation versus selection/control. Therefore, instead of looking at an overall effect of tDCS, one can assess whether the increased excitability offered by tDCS is overall positive versus negative depending on the lexical mechanisms (activation *vs* selection) primarily required by the task. A crucial feature of our investigation will be to look at these potential differences.

The interplay of lexical activation and selection in word retrieval is well demonstrated with paradigms where the presence of semantically related words increases the need for mechanisms of selection and results in longer time/less accuracy in retrieving the target word. This so-called *semantic interference effect* is demonstrated when: a) naming pictures in the presence of semantically related versus unrelated words (*picture-word interference*; Abdel Rahman and Melinger, 2007, Belke and Stielow, 2013, Levelt et al., 1999, Mahon et al., 2007), b) repeatedly naming sets of semantically related versus unrelated words (*cyclic blocked picture naming*; Belke, 2013, Belke and Stielow, 2013, Oppenheim et al., 2010, Schnur et al., 2006), c) comparing naming of exemplars early in a sequence of related pictures – when interference is low – with naming exemplars later in the sequence – when interference has built up (*continuous naming* paradigm; Belke, 2013, Belke and Stielow, 2013, Howard et al., 2006). Effects in picture naming are sometimes compared with effects in reading with the expectation that difficulties with lexical-semantic selection will affect picture naming, but not reading, where targets are retrieved from an orthographic rather than a semantic specification (see Belke, 2008, 2013).

One can put forward different hypotheses on how tDCS could modulate effects of semantic interference. One may assume that *anodal* tDCS, which increases excitability, will improve performance when retrieving words in neutral conditions, but will have more mixed effects when retrieving words in the face of competitors. In this context, effects can even be negative, because it is harder to select among highly activated competitors (i.e., interference effects will *increase*). Furthermore, these contrasting effects may depend on the site of stimulation. It has been suggested that negative effects of anodal tDCS are more likely when applied to temporal areas, which are involved in lexical activation and retrieval (e.g., Indefrey & Levelt, 2004; Piai, Roelofs, Jensen, Schoffelen, & Bonnefond, 2014), while positive effects may be more likely when anodal tDCS is applied to the frontal lobe, which are involved in boosting mechanisms of control and selection (e.g., Hirshorn and Thompson-Schill, 2006, Novick et al., 2010, Scott and Wilshire, 2011). Note, however, that this further hypothesis depends on two controversial assumptions: 1. that effects of tDCS can be focal enough to target specifically one of two adjacent cortical areas (but see Datta et al., 2009); 2. that top-down frontal mechanisms contribute to lexical selection in addition to mechanism of lateral inhibition intrinsic to the lexical module (see Hamilton & Martin, 2005, 2007 for a discussion).

Pisoni, Papagno, and Cattaneo (2012) tested effects of tDCS on semantic interference using a cyclic blocked picture naming paradigm. As predicted, they found increased interference following stimulation of the temporal lobes, but decreased interference following anodal tDCS of the frontal lobe. Meinzer, Yetim, McMahon, and de Zubicaray (2016) and Wirth et al. (2011) also found decreased interference during frontal tDCS with the same paradigm. However, Meinzer et al. (2016) did not replicate the expected increased interference following temporal stimulation and Henseler, Mädebach, Kotz, and Jescheniak (2014) found no significant effect of either frontal or temporal stimulation with a picture-word interference paradigm. These findings, together with more general reviewed findings, point to the limited efficacy of single session tDCS to modulate cognition in healthy participants. In our experimental study, we want to try to replicate these findings, but also explore reasons for variability by considering how tDCS effects may interact with individual differences in cortical excitability.

Participants are likely to differ in baseline levels of cortical excitability for a variety of factors (for extensive reviews, see Krause et al., 2013; Li, Uehara, & Hanakawa, 2015). If cognitive performance depends on an optimum level, with worse performance associated with either too low or too high excitability, then some individuals may show improvement after anodal tDCS, whilst others may show no effect or even worse performance depending on baseline levels. Individual variability in response to both TMS (Silvanto et al., 2008) and tDCS (López-Alonso et al., 2014, Wiethoff et al., 2014) has been demonstrated in the motor domain. López-Alonso et al. (2014), for example, reported that following tDCS more than half of participants showed no increase in TMS-elicited MEPs, but actually a slight decrease. There are also indications that tDCS effects may depend on baseline level of performance (Hsu et al., 2014, Tseng et al., 2012). For example, Tseng et al. (2012) showed that anodal tDCS induced improvements in visual short-term memory and associated increases in event-related potentials (ERPs), but that both of these changes were limited to participants with initially poor performance. These individual sources of variability may compound task-mediated variability in producing variable tDCS outcomes.

In our experimental investigation, we will use naming and reading tasks to assess effects of tDCS both overall and, more specifically, on interference effects. We will use ‘best practice’ anodal stimulation protocols. With cyclic blocked naming picture, we will target frontal areas; with continuous naming, we will contrast stimulation of frontal and temporal areas. Frontal stimulation may be particularly helpful to reduce interference effects, boosting selection mechanisms which control the activation of potential competitors. Temporal stimulation, instead, may increase the activation of competing items, leading to even stronger interference.

In addition, we will consider the possibility of individual variation. Individuals with high baseline levels of excitability may be more likely to exceed an optimal level of activation, especially in naming conditions where a sequence of competitors increases overall activation levels. To evaluate potential effects of tDCS which may have a different sign (positive or negative) in different individuals, we will consider absolute (independent of sign) inter-session differences in an experimental group, where one session is carried out with real stimulation and one with sham stimulation. We will, then, compare these differences with absolute inter-session differences in a control group, where both sessions are carried out in neutral, no stimulation conditions. If tDCS has any effect, differences in the experimental group, due to tDCS, should be larger than differences in the control group, due to random variability between sessions.

Finally, we will also look at effects of tDCS depending on item variability. We will carry out so-called Vincentized analyses where the RTs of each participant are separated into different bins according to their relative speed (very slow, slow, fast, very fast; for a similar method, see Henseler et al., 2014) and then assess the effects of tDCS for each bin. RTs in the ‘very slow’ category may be particularly susceptible to modulation by tDCS (see also Ross, McCoy, Wolk, Coslett, & Olson, 2010).

## 2 Method

### 2.1 Experiment 1: continuous picture naming and reading

Experiment 1 assessed effects of tDCS on picture naming by applying anodal tDCS to frontal (Experiment 1A and 1B) or temporal areas (Experiment 1C). Following Pisoni et al.'s (2012) logic, we expected frontal anodal tDCS to facilitate naming by boosting the ability to select the target word amongst competitors, but temporal stimulation to have possible negative consequences by increasing competition among related items. Differently from Pisoni et al. (2012), however, we used a continuous naming task where participants are presented with sequences of semantically related pictures, but are generally not aware of relationships between pictures because items belonging to the same semantic category are intermixed with distractors. This makes the disruptive effect of competitors less susceptible to strategic control. A reliable increase of RTs for every new item belonging to the same category in a sequence has been shown across studies (with increases of as much as 30 msec for every additional picture; e.g., Belke, 2013, Belke and Stielow, 2013, Howard et al., 2006).

We paired picture naming tasks with corresponding reading tasks to see whether interference effects were specific to the semantic domain and to test more general facilitation effects in word production. If tDCS selectively modulates interference effects in picture naming, with no interference effects in reading, this will show that there are specific effects of tDCS on lexical-semantic control.

#### 2.1.1 Experiment 1A

##### 2.1.1.1 Tasks

Participants carried out *word reading* and *picture naming* tasks, with picture names corresponding to the words used in reading. Stimuli were presented one by one on a computer screen, and participants named stimuli as fast and as accurately as possible. In both tasks, the experimental pictures/words belonged to sets of semantically related items, with related items being separated by a variable number of unrelated items. We measured general speed and accuracy of performance, but also accumulation of semantic interference effects across sets of related pictures.

##### 2.1.1.2 Design

Each participant carried out both tasks in each of two testing sessions, scheduled one week apart and involving parallel versions of the same tasks. In the experimental group, sham stimulation was applied in one session and real stimulation in the other. In the control group, no stimulation was applied in either session. Reading was always done first in order to prime and, therefore, facilitate retrieval of picture names. The order of real and sham stimulation sessions, and which particular version of the task was paired with each session, was counterbalanced across participants. Reading lasted for 5–6 min and picture naming for 9–10 min. Stimulation covered all testing times. It started at the beginning of the reading task, and was applied continuously with no gap when the task was changed.

##### 2.1.1.3 Stimuli

165 colored pictures (720 × 540 pixel dimensions) were taken from a variety of sources, and the same number of corresponding words made up the stimuli. 120 stimuli were experimental and 45 were “fillers”. Experimental stimuli were drawn from 24 semantic categories, with 5 members to each category (for a listing see Appendix A). Presentation of stimuli followed Howard et al. (2006): the first and last five items were filler items; pictures from the same category were presented in a sequence that separated category members by 2, 4, 6, or 8 items composed of fillers or pictures from other categories; each of the 24 categories used a different sequence of lags. The parallel versions of the tasks included the same categories, but different items. To make sure that positional effects were not confounded with other variables, items in different positions were carefully matched for typical age of acquisition (Kuperman, Stadthagen-Gonzalez, & Brysbaert, 2012), frequency (based on CELEX Database; Baayen, Piepenbrock, & Gulikers, 1995), word length and name agreement^1^. These variables were also matched across the two versions of the task (Appendix B).

##### 2.1.1.4 Task procedure

Participants were verbally instructed to read or name the stimuli as fast and as accurately as possible, and to use sub-ordinate nouns (e.g., correct responses to water-lily could be “water-lily” or “lily” but not “flower”). A practice task familiarized participants with the voice key.

Each naming/reading trial started with the presentation of a fixation cross for 1000 msec followed by a blank screen for 250 msec. Stimuli were then presented centered, for 2500 msec or until the participant made a response. A blank screen followed for 500 msec before the next trial started. Stimuli were presented using E-Prime 2 Software and a Dell Laptop computer screen (screen size: 15.6″). Words were presented in Arial typeface 24-font. Vocal responses were recorded using a Sony ICDPX333.CE7 voice recorder. The voice key was a serial response box (Refresher Detector System, Psychology Software Tools, INC). The microphone was a Sony ECM-MS957.

##### 2.1.1.5 tDCS

tDCS was administered using a battery driven NeuroConn DC-Stimulation via a pair of saline soaked sponges. Stimulation was administered using a double-blind procedure, whereby both the experimenter and the participant were unaware of the type of stimulation administered in a given session. For sham stimulation, an intermittent current of 110 μA was delivered for a period of 3 msec every 550 msec. This produces the perceptual sensations of real stimulation without modulating underlying brain areas (Palm et al., 2013). For real stimulation, a constant current of 1 mA was administered for 15 mins with a ramp up and ramp down of 30 sec to reduce discomfort and perceptual differences with sham stimulation. The active electrode (9 cm^2^; current density = .11 mA/cm^2^) was placed over the *left inferior frontal gyrus* (LIFG) whilst the reference electrode (35 cm^2^) was placed over the contralateral supraorbital area. The LIFG was located by measuring 2 cm from the corner of the eye towards the preauricular point of the left ear then 3 cm upwards perpendicular from this measurement, which corresponds to F7 using the electroencephalogram (EEG) 10/20 position system (Devlin & Watkins, 2007). At the end of each session, participants completed a feedback questionnaire (see Fertonani, Rosini, Cotelli, Maria, & Miniussi, 2010) to assess the effectiveness of stimulation blinding.

##### 2.1.1.6 Participants

Fifty undergraduate students from Aston University participated for course credits or financial reimbursement, and were assigned to the experimental or control group in a semi-random fashion. Two participants in the experimental group and control group failed to attend the second session due to other commitments. This left eighteen participants (10 female; 21 ± 2.76) in the experimental group and twenty-eight participants (17 female; 23 ± 2.52) in the control group. All participants were right-handed and native English speakers. We excluded volunteers with language impairments, history of migraine, headaches (frequent or severe), skin disorders (e.g., eczema), any adverse experience to previous tDCS, any history of epilepsy or stroke, head/metal implants, any neurological disorders, and any volunteers who had participated in a tDCS or TMS study in the 6 months prior to the current study.

#### 2.1.2 Experiment 1B

As shown later, Experiment 1A returned no evidence of tDCS effects. Therefore, we changed the stimulation protocol to increase the chances of positive effects as detailed below. In all other methodological aspects, Experiment 1B was the same as Experiment 1A.

##### 2.1.2.1 Stimuli

In Experiment 1A, the order of stimuli was the same for each participant. In Experiment 1B, we created 24 different stimuli orders for each of the two matched versions of the naming (and reading) task, with a different sequence of lags for the different semantic categories, but most importantly with a different set of items in the five positions. Each participant was administered one of these 24 versions (for a similar procedure, see Howard et al., 2006). This was to ensure better counterbalancing of items across positions.

##### 2.1.2.2 Procedure

The order of reading and naming tasks was counterbalanced across participants instead of reading always coming first.

##### 2.1.2.3 tDCS

We increased the intensity of the current from 1 mA to 1.5 mA, and increased the size of the active electrode from 9 to 25 cm^2^. These changes were made to reduce current density (e.g., .06 mA/cm^2^ instead of .11 mA/cm^2^); larger electrodes may make the current more uniform and increase cortical excitation (Miranda, Lomarev, & Hallett, 2006). Stimulation duration was increased by 10 mins (total stimulation duration now 25 mins), with a 5 min delay added between the onset of stimulation and the experimental tasks (during which participants read the instructions again from the computer screen) to ensure tDCS effects were fully engaged at task initiation (see Nitsche and Paulus, 2000, Nitsche et al., 2008, Price et al., 2015). We also added 5 mins at the end to ensure that both tasks were covered by stimulation. Two participants in Experiment 1A had completed naming slightly after stimulation offset (these participants were, in any case, excluded from analysis because they failed to show up to the second session).

##### 2.1.2.4 Participants

Thirty-nine undergraduate students from Aston University participated for course credits or financial reimbursement. Data from four participants in the experimental group were lost due to a technical problem. Thus, the final experimental group included twenty participants (12 female; 21 ± 2.92) and the control group twenty-five participants (13 female; 21 ± 3.73).

#### 2.1.3 Experiment 1C

In Experiment 1C, we assessed whether contrasting effects of tDCS would be found with temporal lobe stimulation. In all methodological details, bar those reported below, Experiment 1C was the same as Experiment 1B.

##### 2.1.3.1 tDCS

The active electrode (25 cm^2^) was placed over the *left mid-posterior temporal lobe* area (pMTG) whilst the reference (35 cm^2^) was placed over the contralateral cheek. The pMTG was determined to be at the halfway point between T3 and T5 using the 10–20 International EEG system. We used the contralateral cheek for the reference electrode as it was speculated that by doing so we can avoid current flow through frontal areas, thereby avoiding the difficulty in localizing possible behavioral effects.

##### 2.1.3.2 Participants

Eighteen (13 female; 19.8 ± 2.8) from Aston University participated for course credit or for financial reimbursement. No participants were allocated to the control group as control data from Experiment 1B also applied to 1C.

### 2.2 Experiment 2: cyclic blocked picture naming

In Experiment 2, tested the effects of tDCS on cyclic blocked picture naming. This paradigm has been extensively studied (for a review, see Belke & Stielow, 2013), and positive effects of tDCS have been reported (Meinzer et al., 2016, Pisoni et al., 2012, Wirth et al., 2011). In this paradigm, participants are asked to repeatedly name sets of pictures that are either semantically related or unrelated. There is, initially, a marked facilitation, with reaction times falling in cycle 2 relative to cycle 1, due to practice. The facilitation continues in subsequent cycles, but the magnitude of this facilitation is reduced for sets of semantically related pictures, due to increased interference amongst competitors which counters facilitation effects. Even more than the previous continuous naming task, this task taps into the ability to select between a set of highly activated lexical representations, because the same small set of pictures is presented repeatedly over a number of cycles. Consistent with this view, imaging evidence shows increased prefrontal activity, presumably linked to the effort for selection, during cyclic blocked picture naming (Schnur et al., 2006), and improvement during anodal tDCS stimulation is associated with increased activity in frontal areas (Wirth et al., 2011).

#### 2.2.1 Task

Participants named as fast and accurately as possible sets of six pictures, with pictures presented one at a time and each set presented four times in a row (four cycles). We measured general naming speed and accuracy, and semantic interference as it builds up across repeated cycles.

#### 2.2.2 Design

Participants carried out two testing sessions in different stimulation conditions (real or sham), one week apart, with parallel sets of materials. The order of real and sham stimulation, and the task version coupled with each type of stimulation, were counterbalanced across participants. The task lasted for roughly 20 min. Stimulation began five minutes before participants initiated the task and lasted the entirety of the task. During the 5 min delay, participants read task instructions via a computer screen.

#### 2.2.3 Stimuli

72 black and white line drawings were taken from the Snodgrass and Vanderwart (1980) set. Pictures were grouped into 12 sets of six pictures: half the sets included *semantically related* pictures, the other half included *semantically unrelated* pictures created by selecting one member from related sets (see Appendix C for a listing). Pictures were presented in 4 cycles in different quasi-random orders (i.e., each picture occupied a different ordinal position across the 4 cycles, and the last item of a cycle and the first of the following cycle were never the same). The related/unrelated blocks were also alternated in a quasi-random order to ensure that no more than two blocks of the same type were shown consecutively. The order of stimulus presentation was the same for all participants. The two versions of the tasks included different semantic categories and different items. Items in the two versions were carefully matched for age of acquisition (Kuperman et al., 2012), frequency (based on CELEX Database; Baayen et al., 1995), word length and name agreement (based on *H* statistic from Snodgrass & Vanderwart, 1980; see Appendix D).

#### 2.2.4 Procedure

Participants were given the same instructions as in Experiment 1. Additionally, they were familiarized with the pictures before beginning the experiment. They were first presented with each picture with its name written below, and then with the pictures on their own and asked to name them. An accuracy score of 90% or more was needed to progress to the main experiment.

In the main experiment, each naming block began with a “Get Ready …” message for 4000 msec, followed a blank screen for 1000 msec and then a fixation cross for 1000 msec. The picture was then presented and remained on the screen until the participant gave his or her naming response. The end of each block of pictures was followed by blank screen for 1000 msec, and by an “End of block …” message which requested the participant to “Press any button” to start the next block. Stimuli were presented using E-Prime 2 Software. Vocal responses were recorded using a TASCAM DR-680 digital voice recorder with a Rode NTG 2 Condenser Shotgun Microphone. Vocal response times were measured using a Cedrus SV-1 voice key.

#### 2.2.5 tDCS

The stimulation protocol matched Experiment 1B in every way except that stimulation was administered using a battery driven Eldith DC-Stimulation device (functionally equivalent to the Neuroconn DC stimulator).

#### 2.2.6 Participants

Thirty-two undergraduate students from University of Birmingham participated for course credits or for financial reimbursement. A technical error meant that data from three participants in the experimental group had to be excluded, leaving seventeen participants (12 female; 21 ± 2.40) in the experimental group and thirteen participants (7 female; 22 ± 1.76) in the control group.

### 2.3 Across experiments

#### 2.3.1 Ethical approval

Our experimental investigation was approved by The Ministry of Defense Research Ethics Committee, by the Aston Research Ethics Committee and by the University of Birmingham Ethics Committee. All participants gave written informed consent prior to any testing session.

#### 2.3.2 Scoring

Response accuracy was scored after each testing session. Only near-synonyms (e.g., “Hoover” instead of “vacuum”) were allowed as correct, any other response was scored as incorrect. Incorrect responses were excluded from RT analysis, as well as RTs below 250 msec and above 2.5 standard deviations from the participant mean. For picture naming, we analyzed percentage error rates and RTs. Errors rates were not analyzed for word reading and cyclic blocked naming tasks because they were very low (<5% and <7%, respectively).

#### 2.3.3 Data re-sampling

In the experimental groups, the order of stimulation (i.e., Sham *vs* Real) and the set of stimuli (i.e., A *vs* B) were counterbalanced. So, in the first session, half of the participants received sham whilst the other half received real stimulation, and half of the participants that received either type of stimulation saw stimuli set A whilst the other half saw set B. In the control group – where stimulation was not applied – half of participants saw set A in the first session and B in the second, and vice versa. To make results from the control group comparable with results from the experimental group, we resampled control data to create two pseudo datasets for sessions 1 and 2, so-called *pseudo-sham* and *pseudo-real* so that the order of presentations (session 1 *vs* 2) and stimulus set (A *vs* B) was also counter-balanced across these two sessions.

#### 2.3.4 Data analysis

Data was analyzed with repeated factor ANOVAs (analysis of variance) to assess the effect of condition in the experimental (Real tDCS *vs* Sham) and control (Pseudo-Real *vs* Pseudo-Sham) groups separately. In addition we ran mixed factor ANOVAs, which combined data from both groups, and considered group as a between-participants factor. This provided a more rigorous test. If tDCS were to have an effect, we excepted an interaction between condition and participant group because the experimental group would show a significantly larger effect of condition than the control group – where stimulation was not applied. For these analyses, we report only the condition by group interactions, since the main effect of condition is irrelevant.

## 3 Results

### 3.1 tDCS feedback questionnaire

Participants tolerated stimulation well. None reported adverse effects nor withdrew from the study because of stimulation. Common sensations were pinching, itching, burning and heat, all with mild to moderate intensity. These sensations differed significantly between stimulation conditions for some participants, but not systematically across experiments or conditions. When asked to identify what form of stimulation they received, participants reported to be guessing or using a ‘gut feeling’. Repeated samples *t*-tests showed that correct guesses never exceeded chance level [Exp 1A: *F*(1,17), = .32, *p* = .58, *η*_*p*_^*2*^ = .02; Exp 1B: *F*(1,19) = .32, *p* = .58, *η*_*p*_^*2*^ = .02; Exp 1C: *F*(1,17) = .14, *p* = .72, *η*_*p*_^*2*^ = .01; Exp 2: *F*(1,16) = 1.00, *p* = .33, *η*_*p*_^*2*^ = .02].

### 3.2 Overall effects of tDCS

**Fig. 1:**
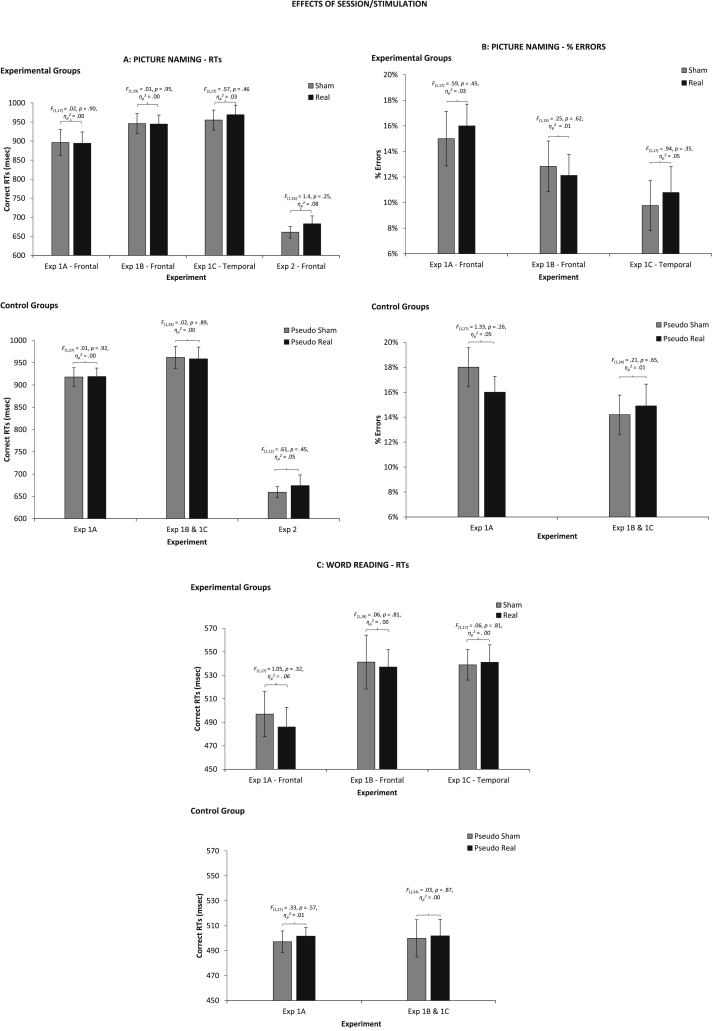
Average correct RTs and accuracy differences between stimulation conditions (Sham *vs* Real for experimental group; Pseudo Sham vs Pseudo Real for control groups across experiments) across experiments. Error Bars indicate Standard Error.

Effects of stimulation across tasks, experiments and participant groups are shown in Fig. 1. We carried out individual one-way ANOVAs for each experiment and participant group to assess whether there was an effect of *Condition* (*Real vs* *Sham* for experimental group; *Pseudo-Real vs* *Pseudo-Sham* for control group). In no experiment was there a significance effect of *Condition*, either in picture naming RTs (across groups: *F* < 1.4, *p* > .25, *η*_*p*_^*2*^ < .08), errors (*F* < 1.33, *p* > .26, *η*_*p*_^*2*^ < .05), or reading RTs (*F* < 1.05, *p* > .32, *η*_*p*_^*2*^ = .06), see Fig. 1.

Mixed factor ANOVAs combined results across experiments and participant groups, with *Group* (*Experimental vs* *Control*) and *Task* (*Continuous Naming vs* *Cyclic Blocked Naming*) as between-participants factors and *Condition* (*Real vs* *Sham* for experimental group; *Pseudo*-*Real vs* *Pseudo*-*Sham* for control group) as a within-participants factor. For picture naming RTs, there was no main effect of *Group* [*F*(1,135) = .002, *p* = .97, *η*_*p*_^*2*^ = .00], but a significant main effect of *Task* [*F*(1,135) = 154.55 *p* < .001, *η*_*p*_^*2*^ = .53], with faster RTs in cyclic blocked naming, as expected. There were no significant interactions, including *Group* × *Task* [*F*(1,135) = .05, *p* = .83, *η*_*p*_^*2*^ = .00], *Condition* × *Task* [*F*(1,135) = 1.1, *p* = .30, *η*_*p*_^*2*^ = .01] and, crucially, *Condition* × *Group* [*F*(1,135) = .12, *p* = .73, *η*_*p*_^*2*^ = .00] or *Condition* × *Group* × *Task* [*F*(1,135) = .01, *p* = .93, *η*_*p*_^*2*^ = .00]. For picture naming errors, *Task* was not a factor because there were not enough errors to analyze in cyclic blocked naming. There was a main effect of *Group* [*F*(1,107) = 8.46, *p* = .004, *η*_*p*_^*2*^ = .07], with the control group being more error prone than the experimental group (*M* ± *SE*: 16 ± 1% *vs* 13 ± 1%). There was, crucially, no *Condition* × *Group* interaction [*F*(1,107) = 1.76, *p* = .19, *η*_*p*_^*2*^ = .02]. For reading RTs, there was a main effect of *Group* [*F*(1,107) = 4.00, *p* = .05, *η*_*p*_^*2*^ = .04] with the experimental group being slower than the control group (524 ± 8 *vs* 500 ± 9), but no *Condition* × *Group* interaction [*F*(1,107) = .52, *p* = .47, *η*_*p*_^*2*^ = .01].

These results show no systematic effects of tDCS. There were some significant differences between the experimental and control group. The experimental group was faster in naming, but slower in reading than the control groups. It is possible that stimulation (both real and sham) modulates level of performance, but more detailed interpretations are difficult.

#### 3.2.1 Interaction with cortical loci of stimulation

To test for a possible interaction between stimulation site and tDCS, for the experimental group only we conducted a mixed factor ANOVA, with *Site* (*Temporal vs Frontal*) as a between-participants factor and *Condition* (*Real vs* *Sham*) as a within-participants factor. We report, here, only experiments 1B and 1C, which used exactly the same paradigm. There were no main effects of *Site* [naming: *F*(1,36) = .25, *p* = .62, *η*_*p*_^*2*^ = .01; errors: *F*(1,36) = 1.71, *p* = .20, *η*_*p*_^*2*^ = .05; reading: *F*(1,36) = .001, *p* = .97, *η*_*p*_^*2*^ = .00] and *Condition* [naming: *F*(1,36) = .26, *p* = .62, *η*_*p*_^*2*^ = .01; errors: *F*(1,36) = .07, *p* = .79, *η*_*p*_^*2*^ = .00; reading: *F*(1,36) = .01, *p* = .92, *η*_*p*_^*2*^ = .00], nor a S*ite* × *Condition* interaction [naming: *F*(1,36) = .36, *p* = .55, *η*_*p*_^*2*^ = .01; errors: *F*(1,36) = 1.01, *p* = .32, *η*_*p*_^*2*^ = .03; reading: *F*(1,36) = .10, *p* = .75, *η*_*p*_^*2*^ = .00].

#### 3.2.2 Direction-neutral effects of stimulation

Here, we considered tDCS effects when allowing for possible opposite outcomes across participants. We found that both participant groups were equally likely to improve or worsen performance relative to sham (or pseudo-sham), with both picture naming RTs [improve:worsen: 37:29_control_ *vs* 35:38_experimental_; χ(1) = .34, *p* = .34], errors [30:23_control_; 29:27_experimental_; χ(1) = .26, *p* = .61], and reading RTs [22:31_control_; 31:25 _experimental_; χ(1) = 2.09, *p* = .15].

**Fig. 2:**
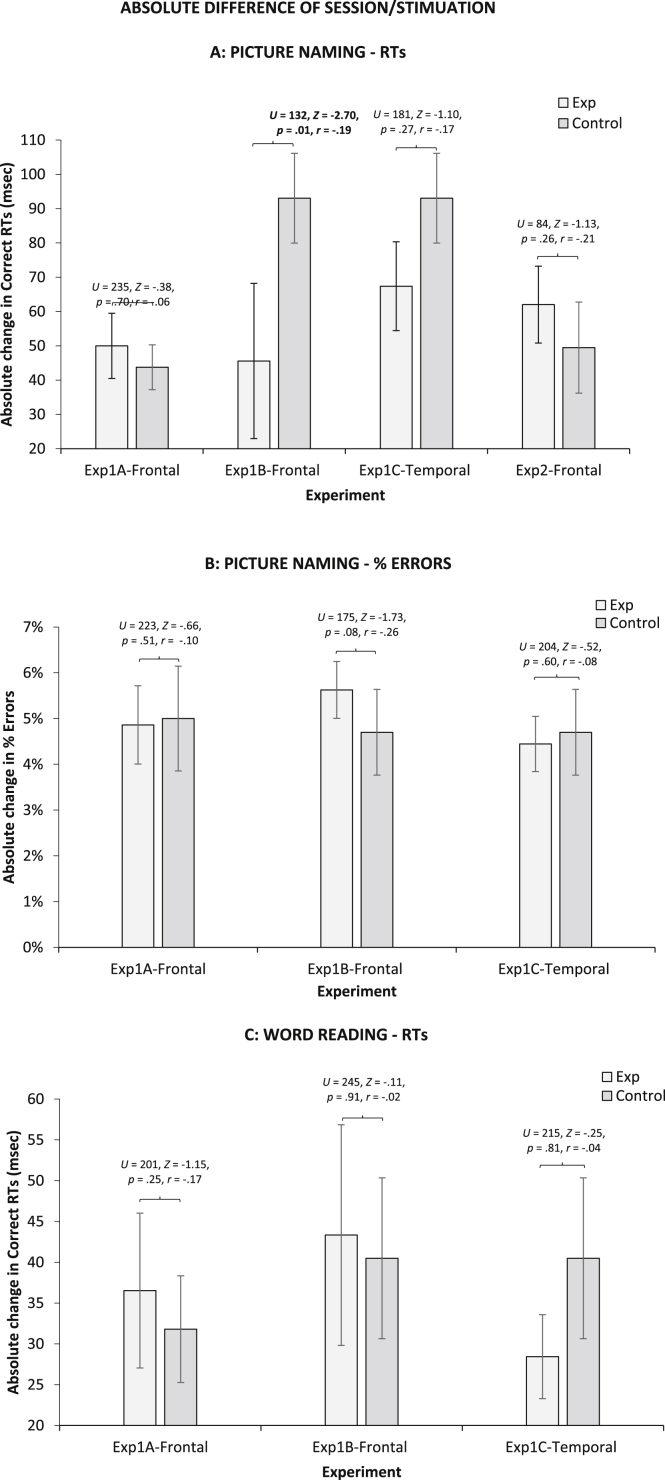
Absolute average correct RTs and accuracy differences between stimulation conditions (Sham *vs* Real for experimental group; Pseudo Sham vs Pseudo Real for control groups) across experiments. Error Bars indicate Standard Error.

We also compared absolute differences between conditions in the experimental and control group via a series of Mann–Whitney *U* tests (as values were non-normally distributed). Results are shown in Fig. 2. Overall, for picture naming RTs, the difference between conditions was smaller in the experimental group relative to the control group (*M* ± SE: 56 ± 6 *vs* 64 ± 7 msec). This was the opposite of what was expected. It could be that stimulation (both real and sham) reduces variability by increasing arousal and/or motivation. It has to be noted however, that this effect was inconsistent with naming errors (5 ± .4 *vs* 5 ± 1%) and reading RTs (37 ± 5 *vs* 36 ± 5 msec).

### 3.3 Effects of tDCS on semantic interference

#### 3.3.1 Cumulative interference

**Fig. 3:**
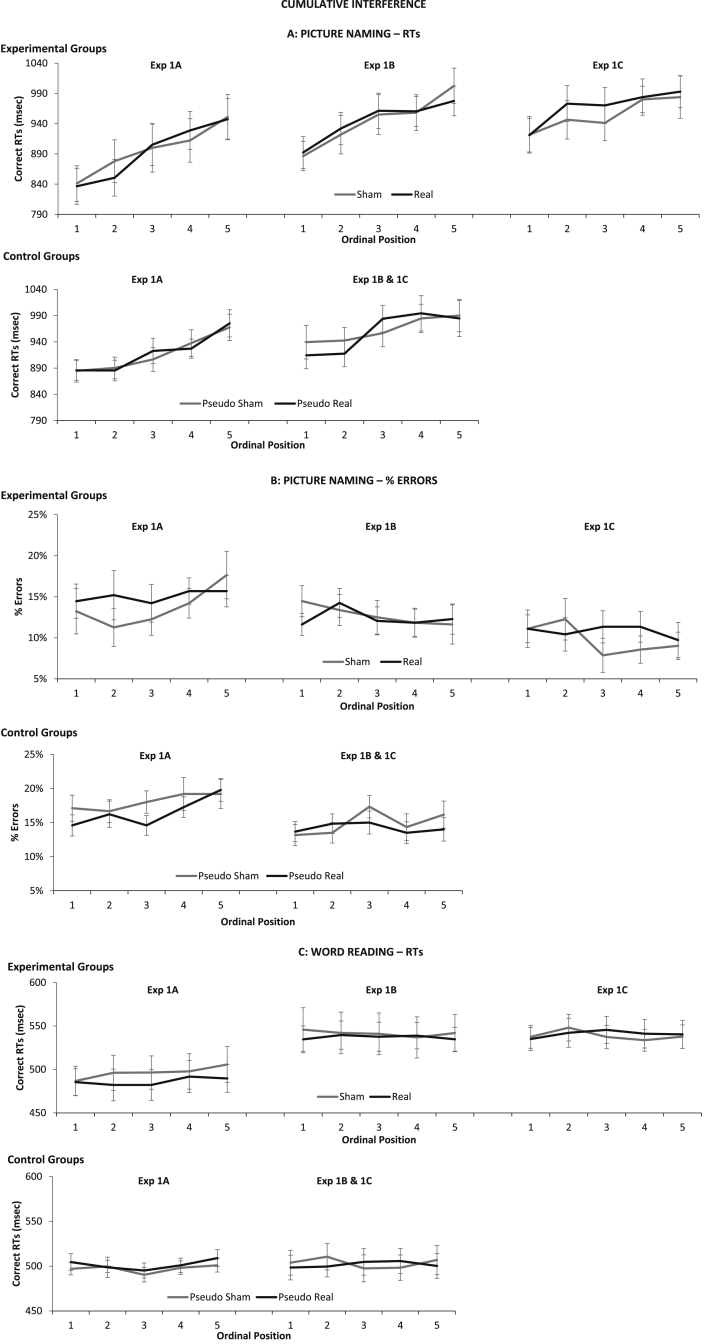
Cumulative semantic interference effect. Average correct RTs and accuracy for ordinal positions across experiments and tasks. Error Bars indicate Standard Error.

Performance across ordinal positions within sets of related items are shown in Fig. 3. Across participant groups, tasks and conditions, our behavioral manipulation worked well. Picture naming shows a steady increase in latencies across positions; errors also show an increasing trend or no effect. Reading shows no systematic effect of position. Crucially, however, there are no detectable effects of tDCS – i.e., the increase in RTs with ordinal position was equivalent with or without tDCS. Numerically, performance was faster in real tDCS than sham in reading experiment 1A (with a slight increase across positions similar to picture naming), but this difference is not significant (see below) and the opposite of what was seen in experiment 1B.

We carried out separate repeated factor ANOVAs for each task, experiment and participant group, with *Ordinal Positions* (1–5) and *Condition* (*Sham vs* Real for experimental group; *Pseudo*-*Sham vs Pseudo*-*Real* for control group) as within-participant factors. With picture naming RTs, there was a main effect of *Ordinal Position*, with latencies increasing with each position [Experimental group: _1A_*F*(4,68) = 27.70, *p* < .001, *η*_*p*_^*2*^ = .62; _1B_*F*(4,76) = 13.83, *p* < .001, *η*_*p*_^*2*^ = .42; _1C_*F*(4,68) = 5.27, *p* = .001, *η*_*p*_^*2*^ = .24; Control group: _1A_*F*(4,68) = 20.62, *p* < .001, *η*_*p*_^*2*^ = .43; _1B/C_*F*(4,96) = 9.4, *p* < .001, *η*_*p*_^*2*^ = .28]. There was no main effect of *Position* with errors [Experimental group: _1A_*F*(4,68) = 1.69, *p* = .16, *η*_*p*_^*2*^ = .09; _1B_*F*(4,76) = .84, *p* = .51, *η*_*p*_^*2*^ = .04; _1C_*F*(4,68) = .45, *p* = .77, *η*_*p*_^*2*^ = .03; Control group: _1B/C_*F*(4,96) = 1.3, *p* = .29, *η*_*p*_^*2*^ = .05] except in the control group for Experiment 1A [*F*(4,68) = 3.12, *p* = .02, *η*_*p*_^*2*^ = .10]. In this case, error rates increased after position three. With reading RTs, there was also a main effect of *Ordinal Position* in Experiment 1A [Experimental group: *F*(4,68) = 2.47, *p* = .05, *η*_*p*_^*2*^ = .13; Control group: *F*(4,108) = 3.4, *p* = .01, *η*_*p*_^*2*^ = .11], but not in Experiment 1B or 1C [Experimental group: _1B_*F*(4,76) = .09, *p* *=* .99, *η*_*p*_^*2*^ = .01; _1C_*F*(4,68) = .79, *p* = .54, *η*_*p*_^*2*^ = .04, *η*_*p*_^*2*^ = .11; Control group: _1B/C_*F*(4,96) = .92, *p* = .46, *η*_*p*_^*2*^ = .04]. Crucially, there were no *Ordinal Position* × *Condition* interactions with naming RTs [Experimental group: _1A_*F*(4,68) = .75, *p* = .56, *η*_*p*_^*2*^ = .04; _1B_*F*(4,76) = .51, *p* = .73, *η*_*p*_^*2*^ = .03; _1C_*F*(4,68) = .34, *p* = .85, *η*_*p*_^*2*^ = .02; Control group: _1A_*F*(4,68) = .38, *p* = .83, *η*_*p*_^*2*^ = .01; _1B/C_*F*(4,96) = 1.1, *p* = .36, *η*_*p*_^*2*^ = .04], naming errors [Experiment group: _1A_*F*(4,68) = .64, *p* = .64, *η*_*p*_^*2*^ = .04; _1B_*F*(4,76) = .46, *p* = .76, *η*_*p*_^*2*^ = .02, _1C_*F*(4,68) = 1.13, *p* = .35, *η*_*p*_^*2*^ = .06; Control group: _1A_*F*(4,68) = .81, *p* = .52, *η*_*p*_^*2*^ = .03; _1B/C_*F*(4,96) = .63, *p* = .65, *η*_*p*_^*2*^ = .03], or reading RTs [Experimental group: _1A_*F*(4,68) = 1.1, *p* = .38, *η*_*p*_^*2*^ = .06; _1B_*F*(4,76) = .43, *p* = .78, *η*_*p*_^*2*^ = .02; _1C_*F*(4,68) = .71, *p* = .59, *η*_*p*_^*2*^ = .04; Control group: _1A_*F*(4,108) = .50, *p* = .74, *η*_*p*_^*2*^ = .02; _1B/C_*F*(4,96) = .14, *p* = .97, *η*_*p*_^*2*^ = .01].

A mixed factor ANOVA across all picture naming experiments with *Group* as a between-participant factor and *Ordinal Position* and *Condition* as within-participant factors showed no three way interaction between *Group* × *Condition* × *Ordinal* *Position* [naming RTs: *F*(4,428) = .45, *p* = .77, *η*_*p*_^*2*^ = .04; errors: *F*(4,428) = .95, *p* = .43, *η*_*p*_^*2*^ = .01; reading RTs: *F*(4,428) = .34, *p* = .85, *η*_*p*_^*2*^ = .00].

#### 3.3.2 Interference by relatedness and cycle

**Fig. 4:**
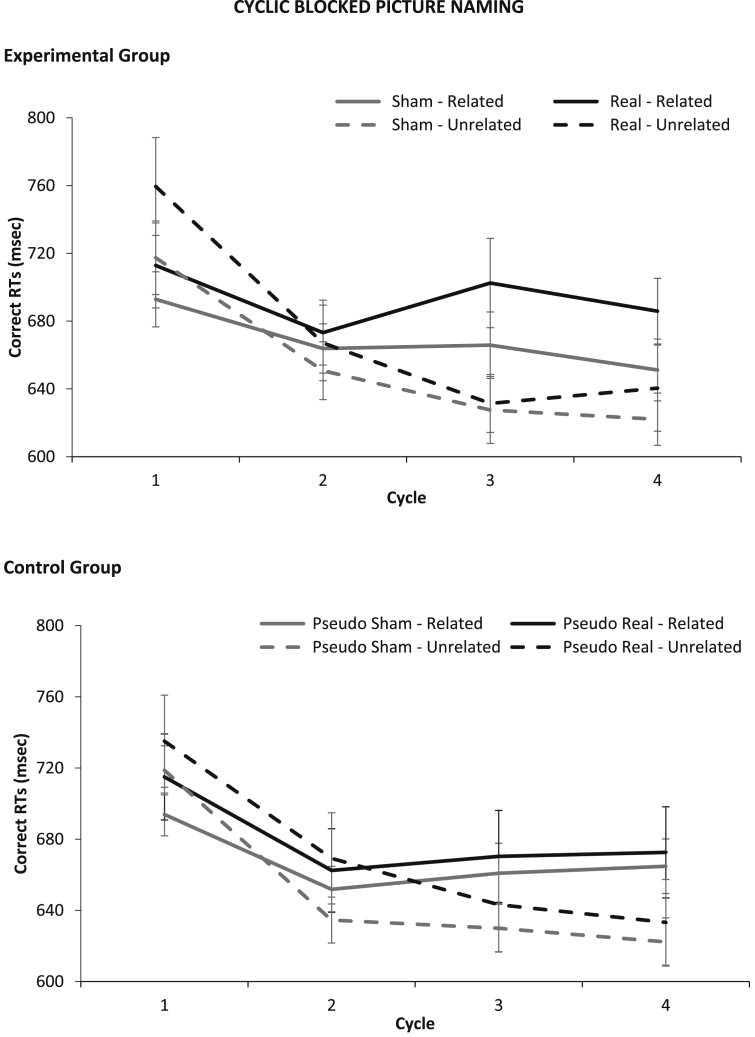
Semantic interference effect by cycle. Average correct RTs for related and unrelated sets across cycles. Error Bars indicate Standard Error.

Results for Experiment 2 are shown in Fig. 4. As expected, semantic relatedness interacted with cycle to modulate performance. For *unrelated* picture sets, participants became progressively faster with every repetition (or cycle), whilst, for *related* sets, naming latencies flattened after initial facilitation between the first and the second cycle. This pattern was produced by both the experimental and control group, and replicates what is typically found with this paradigm (Belke, 2013, Belke and Stielow, 2013).

We carried out a mixed factor ANOVA, with *Group* as a between-participants factor and *Relatedness*, *Cycle* and *Condition* (*Real vs* *Sham* for experimental group; *Pseudo-Real vs* *Pseudo-Sham* for control groups) as within-participants factors. There was a main effect of *Relatedness*, because related sets were slower than unrelated sets [*F*(1,28) = 14.49, *p* = .001, *η*_*p*_^*2*^ = .34], a main effect of *Cycle* [*F*(3,84) = 45.90, *p* < .001, *η*_*p*_^*2*^ = .62], because RTs became faster after the first cycle, and a significant interaction between *Relatedness* × *Cycle* [*F*(3,84) = 28.12, *p* < .001, *η*_*p*_^*2*^ = .50], because related sets were faster than unrelated sets in the first cycle, but then slower. Crucially, there was no main effect of *Group* [*F*(1,28) = .06, *p* = .81, *η*_*p*_^*2*^ = .00], nor a significant interactions between *Group* × *Condition* [*F*(1,28) = .07, *p* = .79, *η*_*p*_^*2*^ = .00], *Group* × *Condition* × *Relatedness* [*F*(1,28) = .98, *p* = .33, *η*_*p*_^*2*^ = .03], *Group* × *Condition* × *Cycle* [*F*(1,28) = .85, *p* = .47, *η*_*p*_^*2*^ = .03, and *Group* × *Condition* × *Relatedness* × *Cycle* [*F*(3,84) = 1.43, *p* = .24, *η*_*p*_^*2*^ = .05].

#### 3.3.3 Aggregated interference

Here, we considered whether tDCS effects are detectable when interference effects are aggregated across conditions. For Experiment 1A–C, we considered the difference in RTs between items in position 4–5 and items in position 1–2. For Experiment 2, we considered the difference between related and unrelated sets at cycle 4 (where the difference should be positive; with related sets being faster) and at cycle 1 (where the difference should be negative; with related sets being slower).

**Fig. 5:**
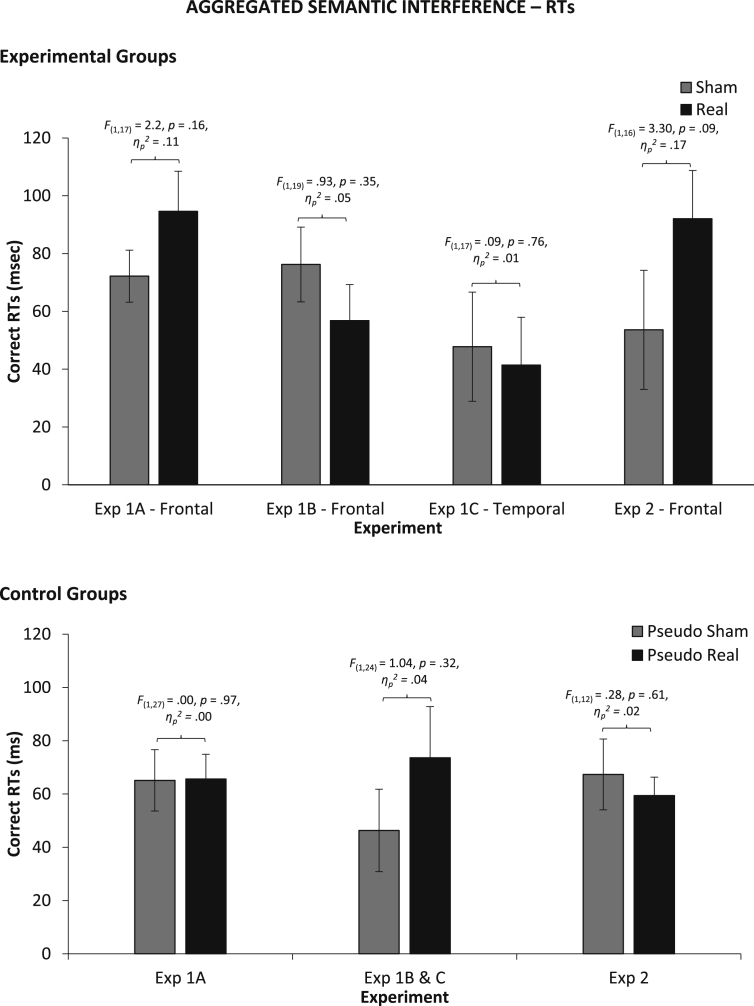
Semantic interference effect averaged across conditions. For experiment 1, interference measured as the differences between the last two and first two ordinal positions; for experiment 2, interference measured as the difference between related and related blocks at cycle 4 versus cycle 1; e.g., (related–unrelated at cycle 4) minus (related–unrelated at cycle 1).

Aggregated interference effects across experiments, groups and conditions are presented in Fig. 5. tDCS clearly had no consistent effect. In the experimental group, interference was larger with tDCS in Experiment 1A and 2, but the opposite was found in Experiment 1B and 1C. We carried out separate one-way ANOVAs for each experiment and participant group, with *aggregate interference* as a dependent measure and *Condition* as a within-participants measure. The results showed no significant main effect of *Condition* (Experimental group: *F* < 3.30, *p* > .09, *η*_*p*_^*2*^ < .17; Control group: *F* < 1.04, *p* > .32, *η*_*p*_^*2*^ < .04). We also carried out a mixed factor ANOVA with *Group* as a between-participants factor and *Condition* as a within-participants factor. Crucially, there was no *Group × Condition* interaction [*F*(1,137) = .01, *p* = .93, *η*_*p*_^*2*^ = .00].

#### 3.3.4 Interaction with cortical loci of stimulation

Given the possibility that tDCS could reduce a semantic interference effect with frontal stimulation, but increase it with temporal stimulation we carried out a mixed factor ANOVA with *aggregate interference* as a dependent measure, *Site* (Frontal-Stimulation-Exp 1B *vs* Temporal-Stimulation-Exp 1C) as a between-participants factor and *Condition* as a within-participants factor. Again, there was no main effect of *Condition* [*F*(1,36) = .80, *p* = .38, *η*_p_^2^ = .022], *Site* [*F*(1,36) = 1.89, *p* = .18, *η*_p_^2^ = .05] and no *Condition × Site* interaction [*F*(1,36) = .21, *p* = .65, η_p_^2^ = .01].

#### 3.3.5 Direction-neutral effects of stimulation

**Fig. 6:**
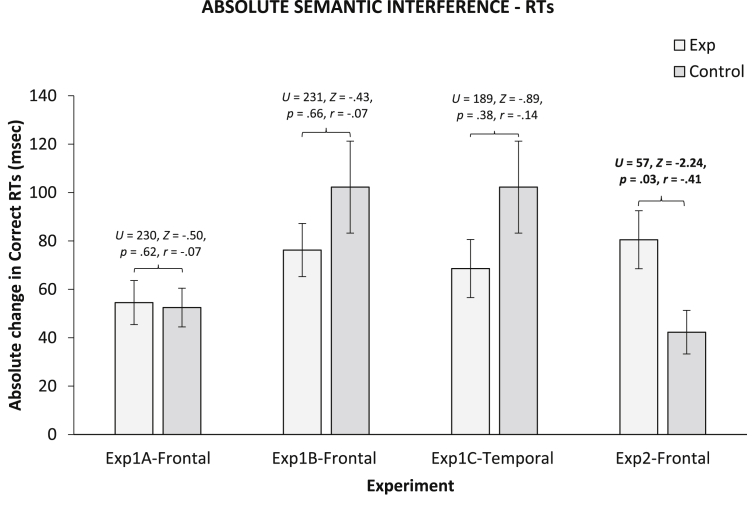
Semantic interference effect in terms of absolute differences in RTs between stimulation conditions (Sham *vs* Real for the experimental group; Pseudo Sham *vs* Pseudo Real for control group) across experiments. Error Bars indicate Standard Error.

Here, we compared absolute differences in interference across stimulation conditions in the experimental and control groups. Results are shown in Fig. 6. Mann–Whitney *U* tests showed that interference effects changed more across conditions in the experimental than in the control group in Experiment 2, but not in any other experiment and effects were numerically in the opposite directions in Experiments 1B and 1C.

#### 3.3.6 Effect of stimulation by magnitude of interference

**Fig. 7:**
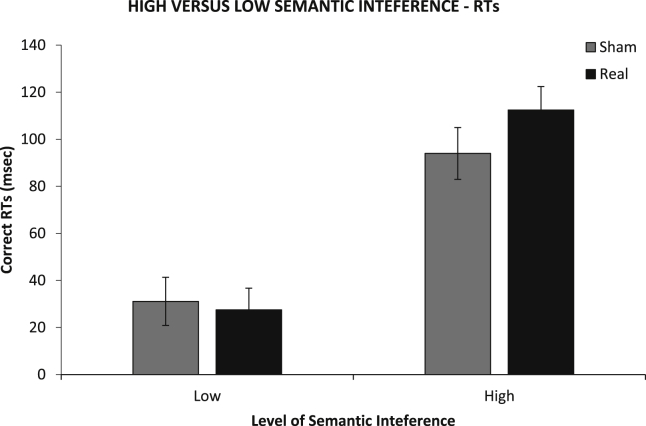
High versus low semantic interference effects effect in terms of RTs across stimulation conditions (Sham *vs* Real for the experimental group; Pseudo Sham *vs* Pseudo Real for control group) and experiments. Error Bars indicate Standard Error.

To assess whether tDCS effects were dependent on the level of semantic interference we grouped experimental participants into those who showed high versus lower levels of semantic interference. We collapsed picture-naming data for all experiments and conducted a median split on the size of semantic interference across both the tDCS and sham conditions. Fig. 7 shows that RTs across participants showing high versus low interference effects were not moderated by stimulation. A mixed factor ANOVA, with *Interference* (High *vs* Low) as a between-participants factor and *Condition* as a within-participant factor showed no significant *Interference* × *Condition* interaction [*F*(1,71) = 1.27, *p* = .26, *η*_*p*_^*2*^ = .02], suggesting that tDCS effects were not moderated by the size of the semantic interference effect.

### 3.4 Effects of stimulation by item difficulty

**Fig. 8:**
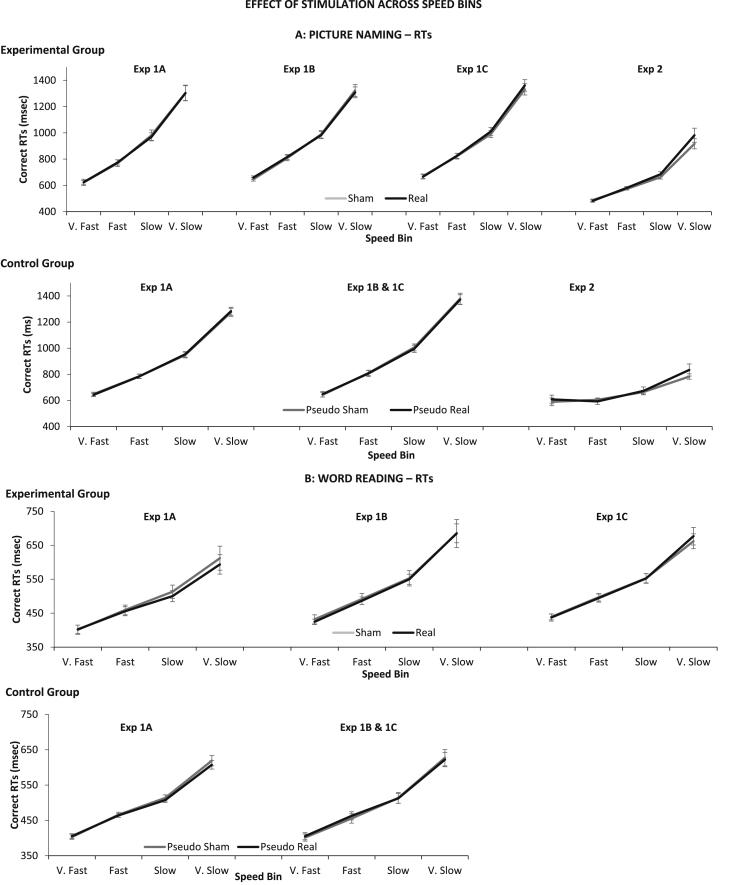
Average correct RTs following Vincentisation. Average RT across speed bins, experiments, and participant groups. Error Bars indicate Standard Error.

We assessed if tDCS effects were limited to items that recruited greater cognitive resources by running a so-called *Vincentisation* analysis. For each task (reading and picture naming), we ranked each participant's RTs within each ordinal position (Experiment 1) or Cycle (Experiment 2), and then placed the RTs into four bins according to speed (e.g., *very slow*, *slow*, *fast*, *very fast*), each with 25% of data. This was done separately for each condition (i.e., Real and Sham; Pseudo-Real and Pseudo-Sham). Results in Fig. 8 show that conditions in the experimental and control groups did not systematically differ depending on speed bin.

We carried out separate mixed factor ANOVAs for each experiment, with *Group* (*Experiment vs* *Control*) as a between-participants factor and *Speed Bin* (1, 2, 3, 4) and *Condition* (Sham *vs* Real for the experimental group; Pseudo-Sham *vs* Pseudo-Real for control group) as within-participants factors. Effects of speed bins are expected and not of interest. Crucially, there was no significant *Speed Bin* × *Group* × *Condition* interaction for picture naming RTs [_1A_*F*(3,132) = .43, *p* = .74, *η*_*p*_^*2*^ = .01; _1B_*F*(3,129) = .14, *p* = .94, *η*_*p*_^*2*^ = .00; _1C_*F*(3,123) = .78, *p* = .51, *η*_*p*_^*2*^ = .02; _2_*F*(3,84) = .43, *p* = .74, *η*_*p*_^*2*^ = .02] or reading RTs [_1B_*F*(3,129) = 1.21, *p* = .31, *η*_*p*_^*2*^ = .03], except for Experiment 1A and 1C [_1A_*F*(3,132) = 3.32, *p* = .02, *η*_*p*_^*2*^ = .07; _1C_*F*(3,123) = 2.68, *p* = .05, *η*_*p*_^*2*^ = .06].

We carried out separate repeated factor ANOVAs to unpack the three-way interaction found in reading RTs for Experiment 1A and 1C, focusing on experimental participants only. We found no significant *Speed Bin × Condition* interaction [_1A_*F*(3,51) = 2.18, *p* = .10, *η*_*p*_^*2*^ = .11; _1C_*F*(3,51) = 1.64, *p* = .19, *η*_*p*_^*2*^ = .09]. Thus, overall, the data showed that tDCS did not selectively modulate performance under high cognitive load.

## 4 General discussion

In the Introduction, we outlined how recent reviews have reported effects of tDCS to be small, inconsistent and not significant when averaged across studies (e.g., Horvath et al., 2015a). Our experimental investigation aimed to provide further evidence for whether tDCS can modulate language processing in normal healthy participants. We carried out four studies with different groups of participants which employed tasks typically used to probe lexical access and word production – namely picture naming and word reading – and used stimulation protocols typically used by studies reporting positive effects (e.g., 1–1.5 mA of anodal stimulation to frontal and temporal areas for 15–25 min during task performance). We made particular efforts to assess whether potential null effects could be masked by variability in the net outcome of tDCS depending on individual baseline levels of cortical excitability and task requirements. We maximized our chances of demonstrating a possible reversal of the advantages generally predicted for language tasks with anodal tDCS of left-hemisphere areas by: 1. Considering task conditions affording a high level of competition from semantically related items, that is, comparing tDCS effects on sets of related versus unrelated items; 2. Considering individual variability in the net outcome of tDCS, that is, assessing whether, with the same task, some participants may show significant facilitation and others significant worsening of performance; 3. Contrasting activation of different areas with the hypothesis that frontal stimulation may boost selection mechanisms, thus reducing interference, while temporal activation may boost lexical activation, thus, increasing interference; 4. Considering preferential effects for participants who demonstrated high semantic interference; 5. Considering possible enhanced/reduced effects of tDCS on difficult to name items. Despite our best efforts, we found no evidence of performance modulation due to the tDCS.

Our results contribute to growing doubts surrounding the reliability of tDCS applied within one stimulation session as a tool to modulate cognition in populations of neurologically intact participants. The effects of tDCS on semantic interference are particularly representative. With temporal stimulation one study found *reduced* interference (Meinzer et al., 2016), one found *enhanced* interference (Pisoni et al., 2012) and two found *no effect* (our own and Henseler et al., 2014). With frontal stimulation three studies found reduced interference (Meinzer et al., 2016, Pisoni et al., 2012, Wirth et al., 2011), but two others found no effect with the same paradigm (our own study) or with a different paradigm (Henseler et al., 2014). Why these differences? A close consideration of the tDCS paradigms employed by these studies does not reveal any clear difference which may be responsible for different outcomes. The three studies which found a reduction of interference effects after frontal stimulation used parameters in the range covered by our experiments. Like us, they stimulated the left inferior frontal gyrus; placed the electrode on the contralateral supraorbital area; used a current density in a similar range (mA/cm^2^ of .029, .057, .080; ours .11–.06); a similar size of the reference electrode 35–100 cm^2^ (our 35 cm^2^), a similar size of active electrode (25–35 cm^2^; our 9–25 cm^2^) and administered the current for a similar duration (20–25 min; ours 15–25 min). Of course, one may always argue that we did not use the right combination of parameters. However, lack of empirical evidence in addition to lack of any appropriate mechanistic model that can provide specific predictions means that we are in the dark when searching for the right parameter combination (for a discussion, see de Berker, Bikson, & Bestmann, 2013; Horvath et al., 2016).

Another possible explanation for our null effects is of course lack of power. Our total samples of 56 and 73 participants for reading and naming respectively allowed us good power to detect medium (.5) or strong (.8) effects of tDCS (1−β > .96) for both. However, the power to detect a *small* effect of tDCS (*effect size* = .25, *α* = .05) was limited even within a within-participants design like ours (1−β = .45 and .56 for reading and naming). To prove or disprove a *small* effect of tDCS with strong statistical power would have required a sample of 128 participants (effect size = .25, 1−β = .8, *α* = .05). This is inconsistent with standards in the field. Most published studies report samples between 10 and 25 participants (see Horvath et al., 2015a, Price et al., 2015 Tremblay et al., 2014). One may want to encourage studies with many more participants, but the fact remains that if effects of tDCS are so small, tDCS is not a tool fit for purpose in the way it is currently employed for modulation of normal cognition. Meta-analyses are of course one way to tackle the issue of small sample sizes. In a review of studies assessing effects of tDCS in reading and picture naming, we pooled studies using a similar protocol to the present study – i.e., applied left anodal tDCS to frontal/temporal lobes – and included the present study. This gave a total sample size of roughly 200 participants. Even with this sample size, we found no evidence of a tDCS effect (see Westwood & Romani, in preparation).

It is possible that future studies will elucidate conditions where single session tDCS is efficacious even in healthy participants. It is also possible, however, that cortical excitability in healthy brains is already close enough to an optimal level that cannot be bettered and/or that homeostatic mechanisms come into play to reduce excessive levels of activation, thus, nullifying any effect of tDCS (Krause & Cohen Kadosh, 2014). Instead, effects of tDCS may only be reliable in neurologically damaged participants where targeted regions may have a pathologically reduced level of excitability (for a review, see Silvanto et al., 2008). A recent review of extant literature on post-stroke aphasia composed of twelve studies (de Aguiar et al., 2015) indicated a general benefit of tDCS across language tasks and types of therapy with varied stimulation protocols. The results showing improvements in picture naming are particularly relevant here (see Fiori et al., 2011; Floel et al., 2011; Kang et al., 2011, Lee et al., 2013, Marangolo et al., 2013, Saidmanesh et al., 2012; but see also Monti et al., 2008).

Alternatively, positive results may be dependent on dose of stimulation (see Meinzer et al., 2014). Positive results with aphasic participants are obtained when tDCS is administered in conjunction with naming once or twice a week for a number of weeks (sessions ranging from 5 to 10). It is possible, therefore, that the key for positive effects of tDCS is not whether the treated population is healthy or impaired, but the stimulation dose and/or repeated application across a number of sessions. It is also possible that positive effects are more likely in tasks that require novel cognitive operations, which are less established in the brain, such as during the acquisition of new processes or representations. Novel operations may be easier to manipulate than operations already well established, such as naming common items (for a similar argument, see Jacobson et al., 2012). It has been shown that tDCS can modify synaptic plasticity by modulating levels of glutamate, GABA, and other neurotransmitters (e.g., dopamine, serotonin, acetylcholine; for extensive reviews, see Medeiros et al., 2012, Stagg and Nitsche, 2011). This may permit modulation of learning. Indeed, a number of studies have shown enhanced learning following repeated stimulation even in normal participants (Cohen Kadosh et al., 2010, Dockery et al., 2009, Meinzer et al., 2014, Reis et al., 2009). Flöel et al. (2008) reported enhanced novel word learning even after a single stimulation session, although the effect vanished after one week.

### 4.1 Conclusions

The bias to publish significant results combined with a lack of appetite for replication (see, Open Science Collaboration, 2015; Vannorsdall et al., 2016), may have given the research community a false sense of tDCS effectiveness. Our results suggest that the unreliability of tDCS results should be taken as a starting point and as a challenge that needs addressing, rather than assuming a level of a reliability that is not there. Across a variety of conditions and analyses, we found no evidence that online tDCS could modulate word retrieval in healthy participants. We performed analyses which considered possible causes of variability, but found no significant results. Further studies should expand on these analyses. Further studies should also assess whether positive effects can be obtained even in healthy participants when stimulation is carried out across different sessions and/or when it involves learning of novel words rather than the modulation of a consolidated vocabulary as in the present study. More generally, our results suggest that the efficacy of tDCS to modulate normal cognition needs to be carefully re-evaluated.

## Funding

The work is funded by the Ministry of Defence and is funded by Defence Science and Technology Laboratory (Dstl), Porton Down, Salisbury, grant DSTLX1000083200. RCM was funded by the Wellcome Trust, grant WT087554.

## Figures and Tables

**Table:** 

	Position										Total	
	1		2		3		4		5		M	SD
	M	SD	M	SD	M	SD	M	SD	M	SD		
**List A**												
Frequency	22	28	17	24	19	38	17	26	19	45	19	33
AoA	6.	3	6	2	6	2	6	2	7	2	6	2
Length	6	2	6	3	6	2	6	2	6.	2	6.	2
Agreement	.7	.9	.9	1.	.8	.7	.6	.9	.7	.7	.7	.9
**List B**												
Frequency	15	20	17	27	18	29	201	38	17	36	18	30
AoA	6	2	6	2	6	2	6	2	6	2	6	2
Length	6	2	6	2	6	3	6	2	7	2	6	2
Agreement	.6	.7	.8	.9	.3	.4	.8	1.	.7	.7	.6	.8

**Table:** 

	Categories													
	Animals		Bugs		Body parts		Clothing		Fruit		Vehicles		Total	Total
	M	SD	M	SD	M	SD	M	SD	M	SD	M	SD	M	SD
**List A**														
Frequency	5.3	1.1	6	1.4	5.1	1.4	5.4	1.7	4.7	.7	5.7	1.7	**5**	**1.3**
Length	5.8	1.2	6.7	3.3	3.8	.8	4.8	1.9	7.2	1.9	6.2	2.6	**5.8**	**2.3**
AoA	4.6	.7	4.5	1.3	3.3	.4	4.5	1.8	4.8	1.2	5	1.7	**4.5**	**1.3**
Agreement *H*	.1	.2	.9	.7	.3	.1	.3	.4	.2	.2	.4	.3	**.4**	**.4**

	Appliances		Birds		People		Tools		Vegetables		Food		Total	
	M	SD	M	SD	M	SD	M	SD	M	SD	M	SD	M	SD
**List B**														
Frequency	6.1	1.2	5.6	1.5	5.2	1.5	5.3	1.4	4.8	1.4	5.2	1.8	**5.4**	**1.4**
Length	6.2	4.6	5.3	1.9	6	1.3	6.2	3.1	6.2	1.7	6.5	1.9	**6.1**	**2.5**
AoA	5.4	2	5.6	1.1	5.7	1.3	6.2	1.5	5.7	1.9	4.1	.8	**5.4**	**1.5**
Agreement *H*	.5	.5	.6	.4	.8	.6	.1	.2	.3	.6	.3	.4	**.4**	**.5**

## Appendix A Stimuli for Continuous Picture Naming/Word Reading. Permissible synonyms in parenthesis. Words in bold represent those presented in one version of the continuous naming task.

***Continuous Picture Naming***

*Birds*: **goose**, **robin, hen (chicken), pigeon, parrot**, rooster (cockerel), duck, pelican, owl, ostrich.

*Body parts*: **tongue, finger, eye, arm, leg**, nail, ear, mouth (lips), foot, nose.

*Buildings*: **shed, barn, lighthouse, church, factory**, cathedral, windmill, skyscraper, tower, castle.

*Clothing items*: **jacket (blazer), socks, sweater (jumper)**, vest, shirt, trousers (chinos), skirt, glove, bathrobe, coat.

*Electrical items*: **headphones, radio, camera, monitor (screen), printer**, laptop, telephone, speaker, mouse, keyboard.

*Farm animals*: **horse**, **bull, lamb, calf, donkey (mule)**, sheep, cow, pig, goat, ox.

*Flowers*: poppy, daffodil, tulip, daisy, dandelion, cactus, sunflower, lavender, rose, lily.

*Fruits*: **kiwi, apple, lemon, strawberry, pear**, pomegranate, orange, cherries, grapes, melon.

*Furniture*: **chest, sofa, armchair, stool (chair), bookcase**, chair, cot, chest, wardrobe, table.

*Insects*: **bee, butterfly, spider, grasshopper (cricket), centipede**, worm, beetle, ant, moth, ladybird.

*Kitchen appliances*: **blender, whisks, washing machine, oven**, **microwave**, dishwasher, food processor, toaster, kettle, hoover.

*Kitchen utensils*: **fork**, **colander**, **cup**, **knife**, **frying pan**, spoon, spatula, glass, bowl, pot.

*Instruments*: **drum**, **guitar**, **flute**, **harp**, **saxophone**, piano, trumpet, violin, clarinet, accordion.

*Landscapes*: **cliffs**, **river**, **mountain**, **lake**, **sea**, beach, waterfall, iceberg, desert, volcano.

*Reptiles*: **crocodile**, **toad**, **turtle**, **python**, **iguana**, frog, cobra, lizard, newt, chameleon.

*Savory food*: **pizza**, **chicken**, **cracker**, **toast**, **steak**, beans, ham, cheese, bacon, hamburger.

*Sea creatures*: **crab**, **starfish**, **eel**, **squid**, **lobster**, prawn (shrimp), clam, octopus, oyster, jellyfish.

*Stationary*: **pen**, **ruler**, **folder**, **paperclip**, **eraser**, pencil, pin, compass, stapler, sharpener.

*Sweet food*: **ice cream**, **marshmallow**, **brownie**, **cake**, **cookie**, doughnut, croissant, muffin, chocolate cheesecake.

*Bathroom items*: **soap**, **perfume**, **bud**, **toothbrush**, **toilet paper**, towel, razor, comb, toothpaste, tweezers.

*Tools*: **axe**, **chisel**, **shears**, **pliers**, **drill**, shovel, mallet, screwdriver, clamp, hammer.

*Vegetables*: carrot, onion, tomato, lettuce, cauliflower, asparagus, potato, pepper, cucumber, celery.

*Transpor*t: tram, bicycle, plane, tractor, caravan, train, bus, van, helicopter, motorbike.

*Safari animals*: **hippopotamus**, **camel**, **kangaroo**, **giraffe**, **cheetah**, elephant, tiger, lion, rhino, zebra.

*Fillers*: nail polish, chain, sword, paintbrush, hourglass, earrings, bag, bauble, slingshot, match, watering can, binoculars, pillow, brick, coins, dartboard, bow, bottle cap, microscope, mascara brush, cone, spray bottle, clock, suitcase, bat, doll, key, broom, note, brush, ring, chessboard, flyswatter, hose, mousetrap, lighter, bucket, candle, acorn, box, door, peanut, pill, hairband, water bottle.

## Appendix C Stimuli for Cyclic Blocked Naming.

***Cyclic Blocked Naming***.

*Animals*: elephant, monkey, panda, rabbit, tiger, zebra.

*Bugs*: bee, butterfly, fly, grasshopper, mosquito, spider.

*Body Parts*: ear, eye, foot, hand, mouth, nose.

*Clothing*: dress, gloves, hat, sock, tie, trousers.

*Fruit*: apple, banana coconut, grapes, pineapple, melon.

*Vehicles*: ambulance, bicycle, boat, bus, motorbike, train.

*Appliances*: fan, fridges, hoover, phone, tv, washing machine.

*Birds*: duck, owl, peacock, penguin, rooster, swan.

*People*: chef, fireman, nurse, painter, police, teacher.

*Tools*: hammer, hoe, pliers, saw, scissors, screwdriver.

*Vegetables*: carrot, corn, mushroom, onion, potato, spinach.

*Food*: cake, hamburger, ice cream, pizza, popcorn, turkey.

## Footnotes

1 Fifteen undergraduate students were shown the 165 pictures and were asked to name each picture. The experiment was self-paced. Name agreement was measured in terms of the number of different names given to each picture. For example, low name agreement would mean relatively more alternatives, and visa versa.

## References

[bib1] Abdel Rahman R., Melinger A. (2007). When bees hamper the production of honey: Lexical interference from associates in speech production. Journal of Experimental Psychology. Learning, Memory, and Cognition.

[bib2] de Aguiar V., Paolazzi C.L., Miceli G. (2015). tDCS in post-stroke aphasia: The role of stimulation parameters, behavioral treatment and patient characteristics. Cortex.

[bib101] Antal A., Keeser D., Priori A., Padberg F., Nitsche M.A. (2015). Conceptual and procedural shortcomings of the systematic review “Evidence that transcranial direct current stimulation (tDCS) generates little-to-no reliable neurophysiologic effect beyond MEP amplitude modulation in healthy human subjects: A systematic review” by Horvath and Co-workers. Brain Stimulation.

[bib3] Baayen R.H., Piepenbrock R., Gulikers (1995). The CELEX lexical database (CD-ROM).

[bib5] Belke E. (2008). Effects of working memory load on lexical-semantic encoding in language production. Psychonomic Bulletin & Review.

[bib6] Belke E. (2013). Long-lasting inhibitory semantic context effects on object naming are necessarily conceptually mediated: Implications for models of lexical-semantic encoding. Journal of Memory and Language.

[bib8] Belke E., Stielow A. (2013). Cumulative and non-cumulative semantic interference in object naming: Evidence from blocked and continuous manipulations of semantic context. Quarterly Journal of Experimental Psychology (2006).

[bib102] de Berker A.O., Bikson M., Bestmann S. (2013). Predicting the behavioral impact of transcranial direct current stimulation: Issues and limitations. Frontiers in Human Neuroscience.

[bib12] Brunoni A.R., Vanderhasselt M.-A. (2014). Working memory improvement with non-invasive brain stimulation of the dorsolateral prefrontal cortex: A systematic review and meta-analysis. Brain and Cognition.

[bib103] Cappon D., Jahanshahi M., Bisiacchi P., Turner R.S., Paul R. (2016). Value and efficacy of transcranial direct current stimulation in the cognitive rehabilitation: A critical review since 2000. Frontiers in Neuroscience.

[bib13] Cattaneo Z., Pisoni A., Gallucci M., Papagno C. (2016). tDCS effects on verbal fluency: A response to Vannorsdall et al (2016). Cognitive and Behavioral Neurology.

[bib14] Cattaneo Z., Pisoni A., Papagno C. (2011). Transcranial direct current stimulation over Broca's region improves phonemic and semantic fluency in healthy individuals. Neuroscience.

[bib16] Cohen Kadosh R., Soskic S., Iuculano T., Kanai R., Walsh V. (2010). Modulating neuronal activity produces specific and long-lasting changes in numerical competence. Current Biology.

[bib17] Datta A., Bansal V., Diaz J., Patel J., Reato D., Bikson M. (2009). Gyri-precise head model of transcranial direct current stimulation: Improved spatial focality using a ring electrode versus conventional rectangular pad. Brain Stimulation.

[bib18] Dedoncker J., Brunoni A.R., Baeken C., Vanderhasselt M.A. (2016). A systematic review and meta-analysis of the effects of transcranial direct current stimulation (tDCS) over the dorsolateral prefrontal cortex in healthy and neuropsychiatric samples: Influence of stimulation parameters. Brain Stimulation.

[bib19] Devlin J.T., Watkins K.E. (2007). Stimulating language: Insights from TMS. Brain.

[bib20] Dockery C.A., Hueckel-Weng R., Birbaumer N., Plewnia C. (2009). Enhancement of planning ability by transcranial direct current stimulation. Journal of Neuroscience.

[bib22] Fertonani A., Rosini S., Cotelli M., Maria P., Miniussi C. (2010). Naming facilitation induced by transcranial direct current stimulation. Behavioural Brain Research.

[bib23] Fiori V., Coccia M., Marinelli C.V., Vecchi V., Bonifazi S., Ceravolo M.G. (2011). Transcranial direct current stimulation improves word retrieval in healthy and nonfluent aphasic subjects. Journal of Cognitive Neuroscience.

[bib24] Flöel A., Rösser N., Michka O., Knecht S., Breitenstein C. (2008). Noninvasive brain stimulation improves language learning. Journal of Cognitive Neuroscience.

[bib104] Floel A., Meinzer M., Kirstein R., Nijhof S., Deppe M., Knecht S. (2011). Short-term anomia training and electrical brain stimulation. Stroke.

[bib25] Hamilton A.C., Martin R.C. (2005). Dissociations among tasks involving inhibition: A single-case study. Cognitive, Affective & Behavioral Neuroscience.

[bib26] Hamilton A.C., Martin R.C. (2007). Proactive interference in a semantic short-term memory deficit: Role of semantic and phonological relatedness. Cortex.

[bib27] Henseler I., Mädebach A., Kotz S., Jescheniak J. (2014). Modulating brain mechanisms resolving lexico-semantic interference during word production: A transcranial direct current stimulation study. Journal of Cognitive Neuroscience.

[bib28] Heth I., Lavidor M. (2015). Improved reading measures in adults with dyslexia following transcranial direct current stimulation treatment. Neuropsychologia.

[bib29] Hill A.T., Fitzgerald P.B., Hoy K.E. (2015). Effects of anodal transcranial direct current stimulation on working and recognition memory: A systematic review and meta-analysis of findings from healthy and neuropsychiatric populations. Brain Stimulation.

[bib30] Hirshorn E.A., Thompson-Schill S.L. (2006). Role of the left inferior frontal gyrus in covert word retrieval: Neural correlates of switching during verbal fluency. Neuropsychologia.

[bib33] Horvath J.C., Carter O., Forte J.D. (2016). No significant effect of transcranial direct current stimulation (tDCS) found on simple motor reaction time comparing 15 different simulation protocols. Neuropsychologia.

[bib34] Horvath J.C., Forte J.D., Carter O. (2015). Brain stimulation quantitative review finds no evidence of cognitive effects in healthy populations from single-session transcranial direct current stimulation (tDCS). Brain Stimulation.

[bib35] Horvath J.C., Forte J.D., Carter O. (2015). Evidence that transcranial direct current stimulation (tDCS) generates little-to-no reliable neurophysiologic effect beyond MEP amplitude modulation in healthy human subjects: A systematic review. Neuropsychologia.

[bib36] Howard D., Nickels L., Coltheart M., Cole-Virtue J. (2006). Cumulative semantic inhibition in picture naming: Experimental and computational studies. Cognition.

[bib38] Hsu T.Y., Tseng P., Liang W.K., Cheng S.K., Juan C.H. (2014). Transcranial direct current stimulation over right posterior parietal cortex changes prestimulus alpha oscillation in visual short-term memory task. NeuroImage.

[bib39] Indefrey P., Levelt W.J.M. (2004). The spatial and temporal signatures of word production components. Cognition.

[bib40] Jacobson L., Koslowsky M., Lavidor M. (2012). tDCS polarity effects in motor and cognitive domains: a meta-analytical review. Experimental Brain Research.

[bib42] Kang E.K., Kim Y.K., Sohn H.M., Cohen L.G., Paik N.J. (2011). Improved picture naming in aphasia patients treated with cathodal tDCS to inhibit the right Broca's homologue area. Restorative Neurology Neurosciece.

[bib44] Krause B., Kadosh R.C. (2014). Not all brains are created equal: The relevance of individual differences in responsiveness to transcranial electrical stimulation. Frontiers in Systems Neuroscience.

[bib45] Krause B., Márquez-Ruiz J., Cohen Kadosh R. (2013). The effect of transcranial direct current stimulation: A role for cortical excitation/inhibition balance?. Frontiers in Human Neuroscience.

[bib46] Kuperman V., Stadthagen-Gonzalez H., Brysbaert M. (2012). Age-of-acquisition ratings for 30,000 English words. Behavior Research Methods.

[bib47] Lee S.Y., Cheon H.J., Yoon K.J., Chang W.H., Kim Y.H. (2013). Effects of dual transcranial direct current stimulation for aphasia in chronic stroke patients. Annals of Rehabilitation Medicine.

[bib48] Levelt W.J., Roelofs A., Meyer A.S. (1999). A theory of lexical access in speech production. The Behavioral and Brain Sciences.

[bib49] Li L.M., Uehara K., Hanakawa T. (2015). The contribution of interindividual factors to variability of response in transcranial direct current stimulation studies. Frontiers in Cellular Neuroscience.

[bib50] López-Alonso V., Cheeran B., Río-Rodríguez D., Fernández-Del-Olmo M. (2014). Inter-individual variability in response to non-invasive brain stimulation paradigms. Brain Stimulation.

[bib51] Mahon B.Z., Costa A., Peterson R., Vargas K.A., Caramazza A. (2007). Lexical selection is not by competition: A reinterpretation of semantic interference and facilitation effects in the picture-word interference paradigm. Journal of Experimental Psychology. Learning, Memory, and Cognition.

[bib52] Mancuso L.E., Ilieva I.P., Hamilton R.H., Farah M.J. (2016). Does transcranial direct current stimulation improve healthy working memory?: a meta-analytic review. Journal of Cognitive Neuroscience.

[bib53] Marangolo P., Fiori V., Calpagnano M.A., Campana S., Razzano C., Caltagirone C. (2013). tDCS over the left inferior frontal cortex improves speech production in aphasia. Frontiers in Human Neuroscience.

[bib54] Medeiros L.F., de Souza I.C.C., Vidor L.P., de Souza A., Deitos A., Volz M.S. (2012). Neurobiological effects of transcranial direct current stimulation: A review. Frontiers in Psychiatry.

[bib55] Meinzer M., Ja S., Copland D.A., Darkow R., Grittner U., Avirame K. (2014). Transcranial direct current stimulation over multiple days improves learning and maintenance of a novel vocabulary. Cortex.

[bib56] Meinzer M., Yetim Ö., McMahon K., de Zubicaray G. (2016). Brain mechanisms of semantic interference in spoken word production: An anodal transcranial Direct Current Stimulation (atDCS) study. Brain and Language.

[bib105] Miniussi C., Harris J.A., Ruzzoli M. (2013). Modelling non-invasive brain stimulation in cognitive neuroscience. Neuroscience and Biobehavioral Reviews.

[bib57] Miranda P.C., Lomarev M., Hallett M. (2006). Modeling the current distribution during transcranial direct current stimulation. Clinical Neurophysiology.

[bib59] Monti A., Cogiamanian F., Marceglia S., Ferrucci R., Mameli F., Mrakic-Sposta S. (2008). Improved naming after transcranial direct current stimulation in aphasia. Journal of Neurology, Neurosurgery & Psychiatry.

[bib62] Nitsche M.A., Bikson M., Bestmann S. (2015). On the use of meta-analysis in neuromodulatory non-invasive brain stimulation. Brain Stimulation.

[bib63] Nitsche M.A., Cohen L., Wassermann E.M., Priori A., Lang N., Antal A. (2008). Transcranial direct current stimulation: State of the art 2008. Brain Stimulation.

[bib65] Nitsche M.A., Paulus W. (2000). Excitability changes induced in the human motor cortex by weak transcranial direct current stimulation. Journal of Physiology.

[bib67] Novick J.M., Trueswell J.C., Thompson-Schill S.L. (2010). Broca's area and language processing: Evidence for the cognitive control connection. Language and Linguistics Compass.

[bib68] Open Science Collaboration, Nosek B.A., Aarts A.A., Anderson C.J., Anderson J.E., Kappes H.B. (2015). Estimating the reproducibility of psychological science. Science.

[bib69] Oppenheim G.M., Dell G.S., Schwartz M.F. (2010). The dark side of incremental learning: A model of cumulative semantic interference during lexical access in speech production. Cognition.

[bib70] Palm U., Reisinger E., Keeser D., Kuo M., Pogarell O., Leicht G. (2013). Evaluation of sham transcranial direct current stimulation for randomized, placebo-controlled clinical trials. Brain Stimulation.

[bib106] Penolazzi B., Pastore M., Mondini S. (2013). Electrode montage dependent effects of transcranial direct current stimulation on semantic fluency. Behavioural Brain Research.

[bib71] Pisoni A., Papagno C., Cattaneo Z. (2012). Neural correlates of the semantic interference effect: New evidence from transcranial direct current stimulation. Neuroscience.

[bib107] Piai V., Roelofs A., Jensen O., Schoffelen J.-M., Bonnefond M. (2014). Distinct patterns of brain activity characterise lexical activation and competition in spoken word production. PLoS ONE.

[bib73] Prehn K., Flöel A. (2015). Potentials and limits to enhance cognitive functions in healthy and pathological aging by tDCS. Frontiers in Cellular Neuroscience.

[bib75] Price A.R., McAdams H., Grossman M., Hamilton R.H. (2015). A meta-analysis of transcranial direct current stimulation studies examining the reliability of effects on language measures. Brain Stimulation.

[bib77] Reis J., Schambra H.M., Cohen L.G., Buch E.R., Fritsch B., Zarahn E. (2009). Noninvasive cortical stimulation enhances motor skill acquisition over multiple days through an effect on consolidation. Proceedings of the National Academy of Sciences of the United States of America.

[bib79] Ross L.A., McCoy D., Wolk D.A., Coslett H.B., Olson I.R. (2010). Improved proper name recall by electrical stimulation of the anterior temporal lobes. Neuropsychologia.

[bib80] Saidmanesh M., Pouretemad H.R., Amini A., Nilipor R., Ekhtiari H. (2012). Effects of transcranial direct current stimulation (2mA – 20min) in patients with non-fluent aphasia disorder. Canadian Journal on Computing in Mathematics, Natural Sciences, Engineering and Medicine.

[bib81] Schnur T., Schwartz M., Brecher a, Hodgson C. (2006). Semantic interference during blocked-cyclic naming: Evidence from aphasia. Journal of Memory and Language.

[bib82] Scott R.M., Wilshire C.E. (2011). Lexical competition for production in a case of nonfluent aphasia: Converging evidence from four different tasks. Cognitive Neuropsychology.

[bib83] Silvanto J., Muggleton N., Walsh V. (2008). State-dependency in brain stimulation studies of perception and cognition. Trends in Cognitive Sciences.

[bib84] Snodgrass J.G., Vanderwart M. (1980). A standardized set of 260 pictures: Norms for name agreement, image agreement, familiarity, and visual complexity. Journal of Experimental Psychology: Human Learning and Memory.

[bib86] Stagg C.J., Nitsche M.A. (2011). Physiological basis of transcranial direct current stimulation. The Neuroscientist: A Review Journal Bringing Neurobiology, Neurology and Psychiatry.

[bib108] Tremblay S., Lepage J., Latulipe-loiselle A., Fregni F., Pascual-leone A., Théoret H. (2014). The uncertain outcome of prefrontal tDCS. Brain Stimulation.

[bib87] Tseng P., Hsu T.-Y., Chang C.-F., Tzeng O.J.L., Hung D.L., Muggleton N.G. (2012). Unleashing potential: Transcranial direct current stimulation over the right posterior parietal cortex improves change detection in low-performing individuals. Journal of Neuroscience.

[bib90] Vannorsdall T.D., van Steenburgh J.J., Schretlen D.J., Jayatillake R., Skolasky R.L., Gordon B. (2016). Reproducibility of tDCS results in a randomized trial: Failure to replicate findings of tDCS-induced enhancement of verbal fluency. Cognitive & Behavioral Neurology.

[bib109] Westwood, S. J., & Romani, C. tDCS modulation of picture naming and word reading: A meta-review of single sessions of tDCS applied to healthy participants. Manuscript in Preparation. 10.1016/j.neuropsychologia.2017.07.031 28757003

[bib92] Wiethoff S., Hamada M., Rothwell J.C. (2014). Variability in response to transcranial direct current stimulation of the motor cortex. Brain Stimulation.

[bib93] Wirth M., Rahman R.A., Kuenecke J., Koenig T., Horn H., Sommer W. (2011). Effects of transcranial direct current stimulation (tDCS) on behaviour and electrophysiology of language production. Neuropsychologia.

